# Rapid and Sensitive Detection of SARS-CoV-2 Using Clustered Regularly Interspaced Short Palindromic Repeats

**DOI:** 10.3390/biomedicines9030239

**Published:** 2021-02-27

**Authors:** Jen-Hui Tsou, Hongjie Liu, Sanford A. Stass, Feng Jiang

**Affiliations:** 1 Department of Pathology, University of Maryland School of Medicine, 10 South Pine Street, MSTF 7th Floor, Baltimore, MD 21201-1192, USA; jtsou@som.umaryland.edu (J.-H.T.); sstass@som.umaryland.edu (S.A.S.); 2 Department of Epidemiology and Biostatistics, School of Public Health, University of Maryland, College Park, MD 20742-2611, USA; hliu1210@umd.edu

**Keywords:** CRISPR, SARS-CoV-2, COVID-19, nucleic acids, detection

## Abstract

Rapid and accurate detection of severe acute respiratory syndrome coronavirus 2 (SARS-CoV-2) is essential for controlling the pandemic of coronavirus disease 2019. Polymerase chain reaction (PCR)-based technique is the standard test for detection of SARS-CoV-2, which, however, requires complicated sample manipulation (e.g., RNA extraction) and is time-consuming. We previously demonstrated that clustered regularly interspaced short palindromic repeats (CRISPR) could precisely detect Human papillomavirus and somatic mutations of Epidermal growth factor receptor gene and Kirsten rat sarcoma viral oncogene homolog gene in plasma. The objective of this study was to develop CRISPR as a rapid test for sensitive detection of SARS-CoV-2. We first combined reverse transcription-isothermal recombinase polymerase amplification and CRSIPR to detect SARS-CoV-2 in genomic RNA of cells infected with the virus. The CRISPR assay with guide RNA against the M gene of SARS-CoV-2 had a sensitivity of 0.1 copies per µL for detection of the virus. We then used the CRSIPR assay to directly analyze raw SARS-CoV-2 samples. The CRISPR assay could sensitively detect SARS-CoV-2 in one hour without RNA extraction. This assay can be performed at a single temperature and with minimal equipment. The results were immediately visualized either by a UV light illuminator or paper strips. The diagnostic value of the test was confirmed in nasopharyngeal swab specimens. Altogether, we have developed a rapid CRISPR test for sensitive detection of SARS-CoV-2.

## 1. Introduction

Severe acute respiratory syndrome coronavirus 2 (SARS-CoV-2) causes coronavirus disease 2019 (COVID-19) [1]. Quick and precise detection of the deadly virus is essential for facilitating early intervention and treatment, and hence reduces mortality of the disease [2]. Reverse transcription PCR (RT-PCR)-based assays have been developed and used as the standard tests for detecting RNA sequences of SARS-CoV-2 [3]. However, the RT-PCR techniques are complex, expensive, time-consuming, and suited to centralized diagnostic laboratories. Therefore, there is an urgent need to develop a point of care (POC) test for rapid and sensitive detection of SARS-CoV-2. 

Clustered regularly interspaced short palindromic repeats (CRISPR) are a family of DNA sequences found within the genomes of prokaryotic organisms [4]. The CRISPR-associated (Cas) immune system has been applied in molecular biology to target and cleave specific nucleic acid sequences, which are commonly used in gene editing. Recently, several Cas proteins have been shown to activate and unleash nonspecific endoribonuclease activity to cause cleavage of DNA or RNA, and thus provide a novel diagnostic approach for nuclei acid detection [5,6,7,8,9]. For instance, using Cas12a, Chen et al. developed a method termed DNA endonuclease-targeted CRISPR trans reporter (DETECTR) for nucleic acid detection [7]. We recently demonstrated that CRISPR-Cas12a could detect nucleic acids of exogenous viruses, such as human papillomavirus, in raw plasma of cervical cancer patients without requiring RNA extraction [10]. Much like a pregnancy test, this CRISPR-Cas12a-POC is a paper-strip test and can “display results immediately” [10]. We have also demonstrated that the CRISPR-based test can rapidly and sensitively detect endogenous DNA mutations of EGFR and KRAS in plasma and tissue specimens of lung cancer patients [11,12]. Broughton et al. used DETECTR to detect SARS-CoV-2 in RNA extracted from clinical specimens [13]. Furthermore, combining CRISPR-Cas13a with mobile phone microscopy, Fozouni et al. develop a rapid assay for testing SARS-CoV-2 from nasal swab RNA. Ding et al. developed the All-In-One Dual CRISPR-Cas12a (AIOD-CRISPR) assay for one-pot and visual SARS-CoV-2 detection [14]. In addition, Young et al. developed a CRISPR-Cas12b-based test called SHERLOCK Testing in One Pot (STOP) for detecting SARS-CoV-2 [15]. However, the current CRISPR techniques for the detection of SARS-CoV-2 require isolating RNA from specimens, which complicates the procedures and increases the risk of cross-contaminations and transmissions of the virus. In this study, we aimed to develop CRISPR-Cas12a as POC testing for sensitive detection of SARS-CoV-2 by directly targeting specimens without RNA isolation and immediately reading results.

## 2. Materials and Methods

### 2.1. SARS-CoV-2 Samples

We obtained NR-52286 and NR-52349 samples from the BEI Resources (Manassas, VA, USA). NR-52286 sample is comprised of cell lysate and supernatant of Vero E6 cells infected with SARS-CoV-2 and has 3.75 × 10^5^ genome equivalents per µL. NR-52349 sample contains a preparation of inactivated SARS-CoV, Urbani (BEI Resources NR-9547) diluted into lung carcinoma cells (A549; ATCC^®^ CCL-185™) with 5.7 × 10^2^ genome equivalents per µL. Genomic RNA from Influenza A virus (H1N1) Strain A/PR/8/34 (ATCC^®^ VR95DQ™) with 6.6 × 10^5^ genome copies per µL was obtained from the ATCC (Manassas, VA, USA). Control plasmid containing the complete N gene from SARS-CoV-2 and double stranded (ds) S gene and M gene, oligonucleotides of primers and CRISPR RNAs (crRNA) or gRNAs, single stranded DNA-FAM-quencher (ssDNA-FQ), and ssDNA-FAM-Biotin (ssDNA-FB) reporters were synthesized by Integrated DNA Technologies (IDT, Coralville, IA, USA).

RT-recombinase polymerase amplification reaction (RT-RPA). RT-RPA reaction was run for isothermal amplification of RNA targets by using TwistAmp^®^ Basic RT (TwistDx, Maidenhead, UK) according to the manufacture’s instruction. Briefly, 50 µL reaction contained 0.48 µM forward and reverse primers (Table 1), 29.5 µL primer free rehydration buffer, 5 µL RNA, and 14 mM magnesium acetate (MgOAc), and was supplemented with 200 U/µL RevertAid Reverse Transcriptase (ThermoFisher Scientific™, Waltham, MA, USA). The RT-PRA mixture was incubated at 42 °C. To directly target specimens without RNA isolation for detection of SARS-CoV-2, 5 µL of supernatant of Vero E6 cells infected with SARS-CoV-2 was used for RT-RPA. The RT-PRA mixture was incubated using the same protocol as described above.

### 2.2. Detection of SARS-CoV-2 by Using CRISPR-Cas12a

**Figure 1:**
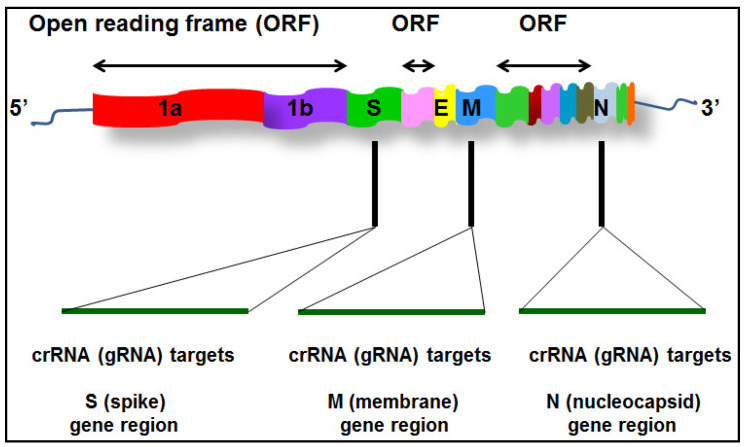
Schematic of severe acute respiratory syndrome coronavirus 2 (SARS-CoV-2) gene structure and locations of three crRNAs (gRNAs) designed to specifically target three genes (S, M, and N) of the virus, respectively.

We first designed guide RNAs (crRNAs, also known as (gRNAs)) specifically to target three genes (S, M, and N) of SARS-CoV-2 (accession NC_045512), respectively (Figure 1 and Table 1). EnGen^®^ LbaCas12a (New England Biolabs, Ipswich, MA, USA) was pre-assembled with each crRNA, respectively, for 30 min and mixed with custom ssDNA-FQ reporter (IDT) (Table 1). The pre-assembled mixture was added directly to 5 µL RT-RPA reactions in a volume 20 µL and incubated at 42 °C on a fluorescence plate reader (Biotek^®^Synergy™ H1, Biotek Instruments Inc., Winooski, VT, USA). Fluorescence kinetics was measured every 10 minutes (λex: 485 nM; λem: 535 nM).

### 2.3. Visual Detection of CRISPR-Cas12a Activity Using a UV Light Illuminator

CRISPR-Cas12a reaction was carried out as described above. The visual detection was based on the reaction solution’s fluorescence signal, in which we captured tubes’ images in the Bio-Rad ChemiDoc™ MP Imaging system (Bio-Rad Laboratories, Hercules, CA, USA) with its built-in UV channel.

### 2.4. Visual Detection of CRISPR-Cas12a Activity Using Lateral Flow Readout

Five µL of amplicon of RT-RPA reaction was combined with 15 µL of LbaCas12a-crRNA complex, ssDNA-FB reporters, and 80 µL of 1× NEBuffer 2.1. The 100 µL of LbaCas12a trans-cleavage assay was incubated at 42 °C. Milena HybriDetect1 later flow dipstick (TwistDx) was directly dipped into the reaction mixture and incubated for 1.5 min. Paper strip was removed, and results were interpreted immediately by photograph using a smartphone camera. A sample with signal significantly increasing on the second line (positive band) and decreasing on the first line (control band) of the strip was considered as a positive result. 

### 2.5. RT-Polymerase Chain Reaction (RT-PCR)

We used the centers for disease control and prevention (CDC)-standard PCR assay targeting the N gene to detect SARS-CoV-2 as previously described [15]. The RT-PCR was performed in RNA isolated from the NR-52286 and NR-52349 samples. Briefly, RNA was amplified with 2019-nCoV CDC EUA KIT (IDT) mixed with GoTaq^®^ Probe One-Step RT-PCR System (Promega) to detect the N2 gene. The one-step RT-PCR assay (20 µL) contained 10 µL GoTaq Probe PCR Master mix with dUTP, 0.4 µL Go Script RT Mix for one-step RT-PCR, 5 µL of the RNA template solution, and 1.5 µL combined Primer/Probe Mix, with final concentration of 500 nM for each primer and of 125 nM for probe in reaction. The thermal cycling protocol included 10 min at 50 °C for reverse transcription, 3 min at 95 °C for inactivation of reverse transcriptase, and initial activation of polymerase, followed by 45 cycles of the two-step cycling (3 s at 95 °C for denaturation, 30 s at 55 °C for annealing and extension). Positive-to-negative cutoff was set at a Ct ≤ 40 for the kit. The assay was conducted in the CFX96 Touch™ Real-Time PCR Detection System (Bio-Rad Laboratories) according to the CDC EUA-approved protocol, and the plate read was set at the annealing and extension step. All samples with a ≤35 Ct value were classified as positive.

### 2.6. Clinical Specimens

Nasopharyngeal swabs of 10 COVID-19 patients and 12 healthy individuals were obtained from Tissue Solutions (Glasgow, UK). The 10 nasopharyngeal swabs of the COVID-19 patients were confirmed as SARS-CoV-2 positive samples, while 12 nasopharyngeal swabs of the healthy individuals were SARS-CoV-2 negative specimens by clinical RT-PCR diagnostic testing. Each sample was collected in 100 µL of M6 viral transport medium (Fisher Scientific, Hampton, NH, USA). To directly analyze the clinical specimens without RNA isolation, 5 µL of each sample in the medium was subjected to RT-RPA. The RT-PRA mixture was incubated using the same protocol as described above in the analysis of supernatant of SARS-CoV-2 samples (NR-52286 and NR-52349). Furthermore, the supernatant of Vero E6 cells infected with SARS-CoV-2 (NR-52286) was tested in parallel as a positive control, while H_2_O was used as a negative control.

### 2.7. Statistical Analysis

We used GraphPad Prism 9 software (Graphpad Inc.; San Diego CA, USA) to perform statistical analysis and plot graphical presentation. Differences in values were evaluated using student *t*-test. *p*-values less than 0.05 were considered as statistically significant in all the analyses. A regression model was used to assess the association between Ct-value and copies of viral RNA/µL and the model-fitting (R2) value.

## 3. Results

### 3.1. CRISPR-Cas12a Can Sensitively Detect SARS-CoV-2

**Figure 2:**
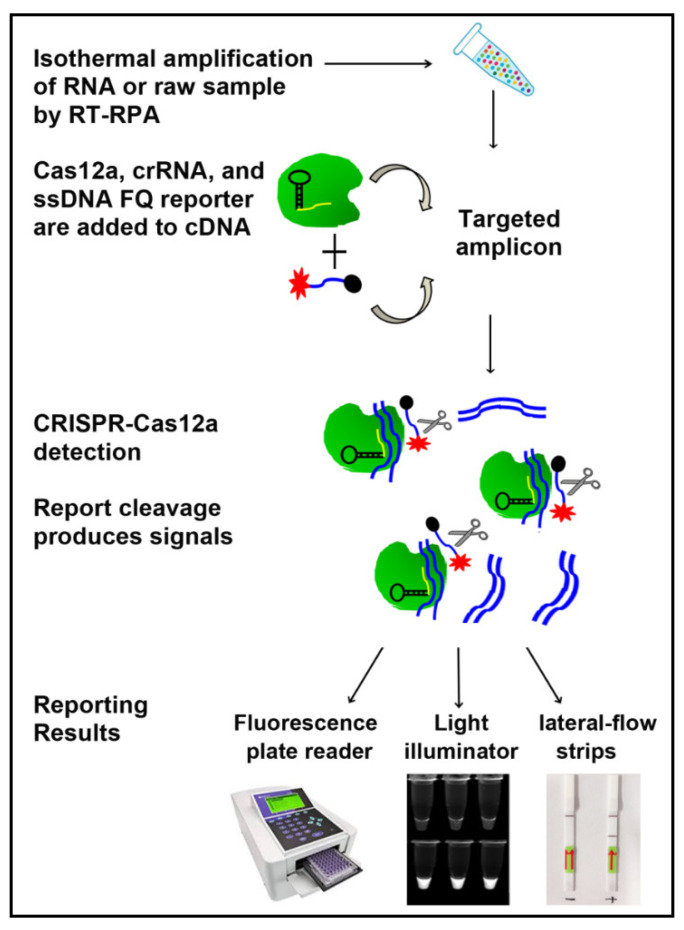
Overview of the clustered regularly interspaced short palindromic repeats (CRISPR)-Cas12a assay to detect SARS-CoV-2. RNA or raw sample was subjected to RT-recombinase polymerase amplification reaction (RT-RPA). The RT-RPA product was mixed with LbaCas12a, CRISPR RNAs (crRNA), and single stranded DNA-FAM-quencher (ssDNA-FQ) reporter. Reaction was performed at 42 °C. Results are read by a fluorescence plate reader, a UV light illuminator, or a paper strip.

As shown in Figure 2, this CRISPR-Cas12a-assay consisted of three steps: (1) RT-RPA of RNA to create dsDNA of the targeted amplicon; (2) the amplified products were incubated with LbaCas12a, specific crRNAs, ssDNA-FQ reporter; and (3) after cleavage of reporter molecules, the detection of the virus was determined and measured by using a fluorescence plate reader, a UV light illuminator, or a paper strip. We designed specific crRNAs to target S, M, and N genes of SARS-CoV-2. The crRNAs guided Cas12a to the target genes and then activated single-stranded DNase activity to cleave the reporter substrates. Whole genomic RNA from cell lysate and supernatant of SARS-CoV-2 samples was tested by CRISPR-Cas12a with the specific crRNAs. Several different concentrations of LbaCas12a, crRNA, and ssDNA-FQ reporter were investigated. The optimized concentrations of LbaCas12a, crRNA, and ssDNA reporter were 640 nM, 640 nM, and 800 nM, respectively.

**Figure 3:**
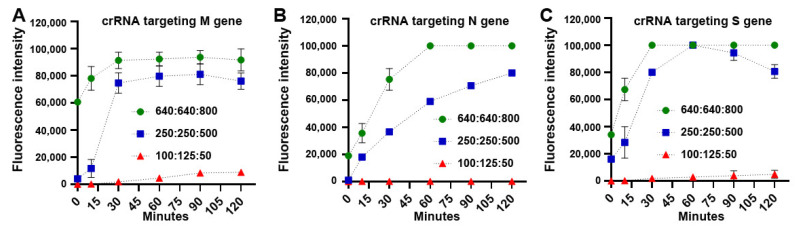
Time courses of fluorescence detection with different concentrations of LbaCas12a: crRNA: ssDNA-FQ reporter to target M (**A**), N (**B**), and S (**C**) of SARS-CoV-2, respectively. The condition of 640 nM LbaCas12a: 640 nM crRNA: 800 nM FAM quencher reporter had significantly increased fluorescence signals for quickly detecting the genes compared with the other conditions.

With the optimized concentrations, fluorescence intensity of CRISPR-Cas12a with the specific crRNAs significantly raised with increase of reaction time in the samples (Figure 3). Furthermore, LbaCas12a with each crRNA could unequivocally identify the corresponding gene of SARS-CoV-2, respectively. Moreover, the crRNA that specifically targeted M gene of SARS-CoV-2 produced more significant fluorescence values in just 5 min, as compared with the crRNAs for N and S genes (Figure 3). Therefore, the crRNA targeting M gene of SARS-CoV-2 might have the highest efficiency for detection of the virus.

**Figure 4:**
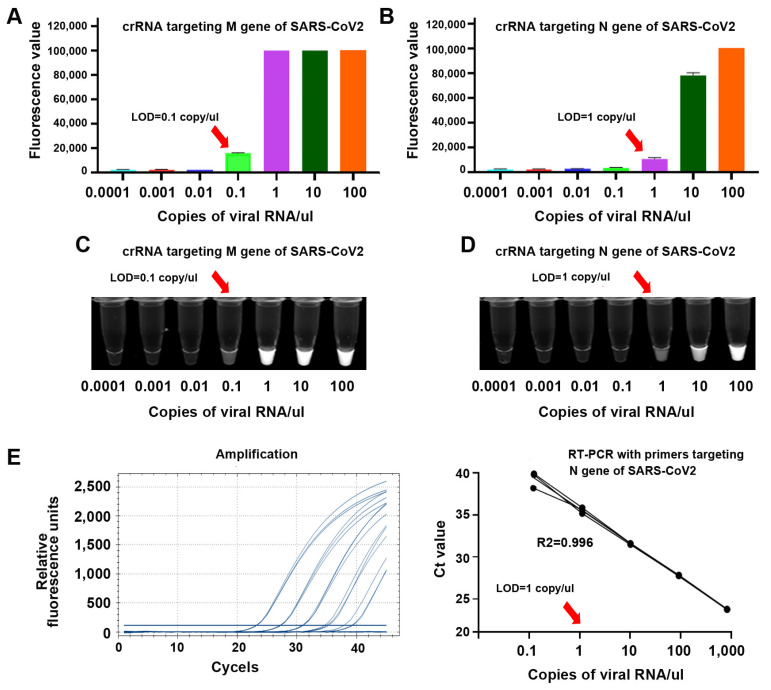
The sensitivity of CRISPR-Cas12a and RT-PCR for detecting SARS-CoV-2 in serially diluted RNA of SARS-CoV-2 samples. When the results were read by a fluorescence plate reader, the limit of detection (LOD) of CRISPR-Cas12a with specific crRNA against M (**A**) and N (**B**) was 0.1 copy/µL/and 1 copy/µL, respectively. When the results were ready by a UV light illuminator, the LOD of CRISPR-Cas12a with specific crRNA against M (**C**) and N (**D**) was also 0.1 copy/µL/and 1 copy/µL, respectively. RT-PCR analysis of N gene of SARS-CoV-2 in the same serially diluted samples had LOD of 1 copy/µL (**E**) for detecting the virus.

To determine sensitivity of CRISPR-Cas12a for detecting the genes, whole genomic RNA of SARS-CoV-2 was serially diluted in nuclease-free water 10-fold and subjected to CRISPR-Cas12a reaction. The results were read by a fluorescence plate reader and a UV light illuminator. For comparison, the samples were also tested by RT-PCR for the detection of N gene of SARS-CoV-2. CRISPR-Cas12a with crRNAs against M and N genes had a limit of detection (LOD) of 0.1 and 1 copy/µL, respectively, for detection of SARS-CoV-2 (Figure 4A,B). The sensitivities of CRISPR-Cas12a were confirmed when the results were directly visualized by a UV light illuminator (Figure 4C,D). RT-PCR with primers against N gene had an LOD of 1 copy/µL (Figure 4E). CRISPR-Cas12a with crRNA (gRNA) against N gene had the same sensitivity, as did RT-PCR targeting the N gene for detecting SARS-CoV-2. Furthermore, CRISPR-Cas12a with crRNA against M gene had a 10-fold higher sensitivity than RT-PCR targeting the N gene for detecting SARS-CoV-2.

### 3.2. CRISPR-Cas12a Can Specifically Detect SARS-CoV-2

**Figure 5:**
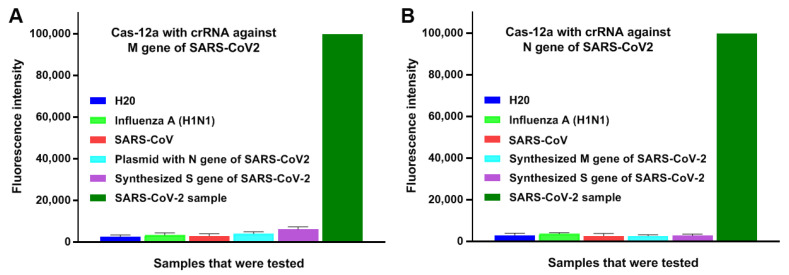
The specificity of CRISPR-Cas12a with the crRNAs for detection of SARS-CoV-2 in different types of samples, including severe acute respiratory syndrome coronavirus (SARS-CoV), SARS-CoV-2, and influenza A viruses. (**A**). The assay with crRNA against M gene of SARS-CoV-2 specifically detected SARS-CoV-2. (**B**). The assay with crRNA against N gene of SARS-CoV-2 particularly detected SARS-CoV-2.

To determine specificity of CRISPR-Cas12a, synthetic dsDNA sequences of S and M genes, plasmids containing N gene of SARS-CoV-2 and genomic RNA of influenza A and SARS-CoV, were subjected to CRISPR-Cas12a. As shown in Figure 5, CRISPR-Cas12a with each specific crRNA against M and N genes could unambiguously identify the corresponding genes, respectively, of SARS-CoV-2. Furthermore, CRISPR-Cas12a with the specific crRNAs against M and N genes of SARS-CoV-2 clearly identified the virus, and did not exhibit positive signals in samples of influenza A and SARS-CoV, which shared a high degree (~82%) of nucleotide identity with SARS-CoV-2 [16] (Figure 5). Therefore, the CRISPR assay possessed high specificity for detecting the targeting gene of SARS-CoV-2 and no cross reactions with other viruses.

### 3.3. CRISPR-Cas12a Can Directly Target Raw Specimens without RNA Isolation and the Results Can Be Immediately Read

**Figure 6:**
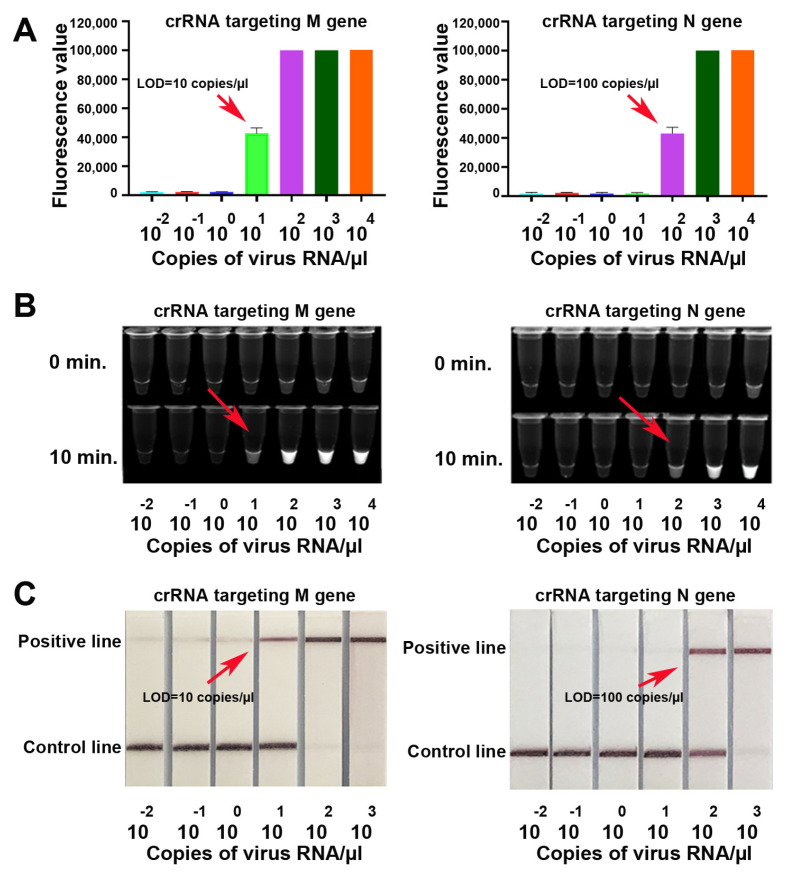
Directly visualizing the results of CRISPR-Cas12a. Heated samples without RNA extraction were subjected to RT-RPA and CRISPR-Cas12a reaction. (**A**). When the results were read in a fluorescence plate reader, CRISPR-Cas12a with specific crRNA against M or N gene detected SARS-CoV-2 with LOD of 10 and 100 copies/µL, respectively. (**B**). When the results were read in a UV light illuminator, CRISPR-Cas12a with crRNA against M or N gene detected SARS-CoV-2 in 10 min with LOD of 10 and 100 copies/µL, respectively. (**C**). When the results were read on paper strips, the CRISPR-Cas12a assay also had LOD of 10 and 100 copies/µL, respectively, for detecting SARS-CoV-2.

We directly added cell lysate and supernatant of SARS-CoV-2 samples into the RT-RPA reaction, from which five µL was then applied to the reaction of CRISPR-Cas12a. The results were read on a fluorescence plate reader. Cas12a with crRNAs specific for M or N gene clearly identified SARS-CoV-2 with a LOD of 10 or 100 copies/µL, respectively (Figure 6A).

As shown in Figure 6B, super-bright fluorescence signal of the positive results could be directly visualized by a UV light illuminator in just 10 min (Figure 6B). Consistently, when the paper strips were used, Cas12a with crRNAs specific for M or N gene clearly identified SARS-CoV-2 with a LOD of 10 or 100 copies/µL, respectively (Figure 6C). Furthermore, from the pretreatment of samples until reading the results, the total turnaround time for the CRISPR-Cas12a-based assay was about one hour without requiring an expensive instrument. In addition, the entire CRISPR-Cas12a process took place at the same temperature (42 °C). Therefore, the CRISPR-Cas12a system could potentially be used as a POC test for rapid detection of SARS-CoV-2.

### 3.4. CRISPR-Cas12a Can Directly Target Clinical Specimens for Detection of SARS-CoV-2

**Figure 7:**
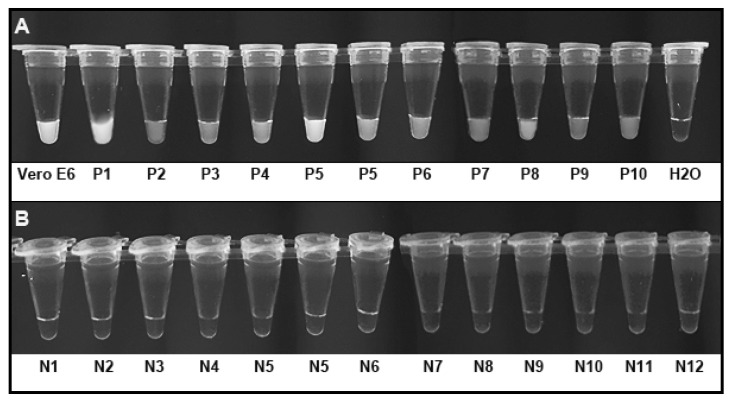
CRISPR-Cas12a could directly test clinical specimens for SARS-CoV-2. Five µL of each clinical sample was heated at 65 °C for 30 min and subjected to RT-RPA without RNA extraction, and then underwent CRISPR-Cas12a reaction. The results were directly visualized by imaging the tubes. (**A**) Ten SARS-CoV-2-positive nasopharyngeal swabs had the evidence of SARS-CoV-2 as demonstrated by bright fluorescence signals. The supernatant of Vero E6 cells infected with SARS-CoV-2 was positive, while H_2_O was negative for SARS-CoV-2. (**B**) The nasopharyngeal swabs from 12 control individuals displayed negative results as demonstrated by the lack of fluorescence signal. Vero E6, Vero E6 cells infected with SARS-CoV-2; P, positive nasopharyngeal swabs for SARS-CoV-2; N, negative nasopharyngeal swabs for SARS-CoV-2.

The CRISPR-Cas12a assay was performed in ten nasopharyngeal swabs of COVID-19 patients and 12 healthy individuals. In each run, the supernatant of Vero E6 cells infected with SARS-CoV-2 was used as a positive control, while H_2_O was used as a negative control. The result in each tube was visualized based on the reaction solution’s fluorescence signal in an imaging system as described above. As shown in Figure 7A, the bright fluorescence signals of the positive results for SARS-CoV-2 in the Vero E6 cells infected with SARS-CoV-2 and ten nasopharyngeal swabs of COVID-19 patients were directly visualized as early as 10 min (Figure 7A). Therefore, the ten SARS-CoV-2-positive samples had the evidence of SARS-CoV-2 determined by the CRISPR-Cas12a system. However, no fluorescence signal was observed in the nasopharyngeal swabs from 12 individuals who had no SARS-CoV-2 infection after the incubation time was extended to 30 min (Figure 7B). Therefore, the nasopharyngeal swabs from 12 control individuals did not have the evidence of SARS-CoV-2 by the CRISPR-Cas12a system.

The positive predictive agreement and negative predictive agreement of the CRISPR-Cas12a assay relative to the clinical RT-PCR diagnostic testing were 100%, respectively, for detection of SARS-CoV-2.

## 4. Discussion

RT-PCR is the most commonly used method for the detection of SARS-CoV-2. However, RT-PCR is time-consuming and labor intensive, limiting its use in point-of-care settings. CRISPR has been investigated to develop tests for detection of SARS-CoV-2 [13,14,15]. However, these CRISPR-based techniques require RNA extraction from specimens, which complicates the procedures and produces contaminations and inconstant results [13,14]. To address the obstacles, herein we combine CRISPR-Cas12a with RT-RPA to directly analyze lysed samples and immediately visualize results using a UV light illuminator or paper strips for detection of SARS-CoV-2. This new CRISPR-Cas12a assay has the following advantages: (1) It can directly target samples for detecting SARS-CoV-2 without requiring the complicated procedures (RNA extraction). The whole process of the test takes about 60 min. (2) The test takes place at a constant temperature of 42 °C, and thus could act as a real one-step detection of SARS-CoV-2 and enable POC testing. (3) The test has at least the same sensitivity as does the standard RT-PCR in genomic RNA of cells infected with the virus, and therefore, might provide a sensitive POC tool for detection of SARS-CoV-2. (4) The clinical significance of the CRISPR-Cas12a assay was confirmed in nasopharyngeal swab specimens of COVID-19 patients and healthy individuals.

Several CRISPR-Cas-based detection platforms, including SHERLOCK (Cas13) and DETECTR (Cas12a), were developed with high sensitivity and specificity [6,17,18]. Recently, the Cas12b system was also shown to cleave dsDNA with high activity and specificity [19,20]. We will compare the different CRISPR-Cas-based detection platforms in a future study for detection of SARS-CoV-2. Furthermore, mutations related to SARS-CoV-2 have been reported that are associated with a more transmissible form of the virus [21,22]. We will also determine if the Cas12a system with specific crRNAs against the mutation could detect related variations.

## 5. Conclusions

The CRISPR-Cas12a-based technique is a promising POC test for detecting, monitoring, and tracking SARS-CoV-2. Nevertheless, it should be validated for diagnostic efficiency in a large and prospective study.

## Figures and Tables

**Table 1:** Sequences for RPA primers, PCR primers/probes, guide RNAs (crRNAs), and reporter substrates.

**Primers/Probe for PCR**	
2019-nCoV_N2-F	TTACAAACATTGGCCGCAAA
2019-nCoV_N2-R	GCGCGACATTCCGAAGAA
2019-nCoV_N2-P	FAM-ACAATTTGC/ZEN/CCCCAGCGCTTCAG-3IABkFQ
**Primers for RT-RPA**	
COVID19 M-RPAF	CTTGATGTGGCTCAGCTACTTCATTGCTTC
COVID19 M-RPAR	TGGAGTGGCACGTTGAGAAGAATGTTAGTTTC
COVID19 N2-RPAF	TGATTACAAACATTGGCCGCAAATTGCACA
COVID19 N2-RPAR	AGGTCAACCACGTTCCCGAAGGTGTGACTT
COVID19 S2-RPAF	TATTCTACAGGTTCTAATGTTTTTCAAACAC
COVID19 S2-RPAR	AGCGCATATACCTGCACCAATGGGTATGTCAC
**crRNAs**	
COVID19 M gRNA	UAAUUUCUACUAAGUGUAGAUCGCGUACGCGUUCCAUGUGG
COVID19 N2 gRNA	UAAUUUCUACUAAGUGUAGAUCCCCCAGCGCUUCAGCGUUC
COVID19 S2 gRNA	UAAUUUCUACUAAGUGUAGAUAUAGGGGCUGAACAUGUCAA
**Reporter Substrates**	
ssDNA-FQ reporter	/56-FAM/TTATTATT/3BHQ_1/
ssDNA-FB reporter	/56-FAM/TTATTATT/3Bio/

Abbreviations: RPA, recombinase polymerase amplification; PCR, real-time polymerase chain reaction; FQ, FAM-quencher and FB, FAM-Biotin; crRNAS, CRISPR RNAs.

## Data Availability

Not applicable.
